# Impact of authoritarian leadership on knowledge hiding: the serial multiple mediation effects of hindrance stressors and job insecurity

**DOI:** 10.3389/fpsyg.2026.1821751

**Published:** 2026-09-24

**Authors:** Bowen Shan, Xiu Jin

**Affiliations:** Department of Business Administration, Gachon University, Seongnam-si, Republic of Korea

**Keywords:** authoritarian leadership, hindrance stressors, job insecurity, knowledge hiding, small- and medium-sized enterprises

## Abstract

**Introduction:**

In an increasingly knowledge driven economy, understanding how authoritarian leadership shapes employees’ knowledge hiding is critical for sustaining organizational learning and adaptability. Drawing on the conservation of resources theory and the Job Demands Resources model, this study examines how authoritarian leadership influences employees’ knowledge hiding behavior through hindrance stressors and job insecurity among employees in small and medium sized enterprises in China. More importantly, this study conceptualizes knowledge hiding as a four dimensional construct encompassing playing dumb, evasive hiding, rationalized hiding, and procrastination.

**Methods:**

Drawing on a sample of 363 full-time employees, we tested a serial mediation model, in which authoritarian leadership increased knowledge hiding through its effects on hindrance stressors and job insecurity.

**Results:**

All the proposed hypotheses received empirical support. The findings demonstrate that authoritarian leadership affects knowledge hiding through interpersonal dynamics and a cascade of stress-based psychological mechanisms.

**Discussion:**

This study highlights the contextual relevance of small- and medium-sized enterprises in China, where hierarchical structures, resource scarcity, and cultural norms amplify employees’ sensitivity to leadership pressure. The practical implications of reducing knowledge hiding and fostering transparent knowledge exchanges are also discussed.

## 1 Introduction

To stay inventive in an increasingly competitive, complex, and dynamic environment, companies worldwide are turning to nontraditional tools, novel approaches, and fresh perspectives to generate, combine, and integrate knowledge and information (Lyu et al., 2024). As organizations increasingly rely on knowledge to maintain competitiveness, employees’ willingness to share knowledge becomes a decisive factor in shaping organizational learning and adaptability (Yuan and Lam, 2024). Owing to the pivotal role of knowledge, extensive research has explored effective approaches to manage employees’ intellectual capital (Zhang et al., 2025). Yet, knowledge hiding—defined as the intentional concealment of knowledge when requested by others—remains a widespread and detrimental phenomenon (Connelly et al., 2012). According to Anand et al. (2022), knowledge hiding occurs when individuals intentionally conceal knowledge requested by others, distinguishing it from related behaviors such as knowledge hoarding, knowledge withholding, and a lack of knowledge sharing. When employees face excessive task demands without sufficient information or capabilities, their work engagement may decline and their willingness to share knowledge may weaken (Nguyen et al., 2022). Moreover, uncertainty about job security may encourage employees to retain valuable knowledge as a means of preserving their competitive advantage (Aarabi et al., 2013). Over time, these unfavorable conditions may foster workplace cynicism and subsequently increase employees’ knowledge hiding (Nguyen et al., 2022). This behavior compromises team collaboration and trust, thereby weakening organizational adaptability in competitive environments. While prior research has explored individual traits and interpersonal dynamics that contribute to knowledge hiding (Liu et al., 2025), contextual and psychological stressors within the work environment may play a pivotal role in triggering such defensive behaviors (Benítez-Núñez et al., 2024; Bao et al., 2024). Recent research by Zhang et al. (2025) demonstrated that knowledge hiding can significantly undermine positive reciprocity outcomes such as creative and innovative performance, task performance, and extra-role behavior, while simultaneously intensifying negative reciprocity outcomes, including turnover intentions and workplace deviance. These findings suggest that employees’ knowledge-hiding behavior not only hampers their performance and collaborative potential but also triggers reciprocal negative responses that may harm overall organizational functioning.

Leadership is one of the core forces shaping these behavioral tendencies. Authoritarian leadership, marked by centralized authority, strict discipline, and limited employee discretion, remains deeply embedded in managerial practices (Farh and Cheng, 2000). Although previous studies have shown that authoritarian leadership can enhance employees’ job performance (Wang et al., 2022; Thawornlamlert et al., 2026), these studies primarily focused on performance-related outcomes. The present study instead examines a potential negative consequence of authoritarian leadership by investigating its influence on employees’ knowledge hiding. Although authoritarian leadership may promote discipline and task execution, particularly under conditions of environmental turbulence, its emphasis on rigid control and unquestioned obedience can also heighten employees’ perceptions of stress, constraints, and threats, thereby triggering defensive behaviors such as knowledge hiding (Chen et al., 2024). Despite increasing scholarly interest in the antecedents of knowledge hiding, existing research has largely emphasized interpersonal and relational factors, including interpersonal distrust, perceived competition, and low-quality leader–member exchange (Connelly and Zweig, 2015; Semerci, 2019; Zhao et al., 2019). However, this focus overlooks broader structural and psychological factors, especially those rooted in leadership style and organizational stress, which may create conditions under which employees are more likely to withhold knowledge (Shaukat et al., 2025). In particular, authoritarian leadership, characterized by centralized power, unilateral decision-making, and limited follower autonomy, undermines psychological safety and fosters defensive silence (Chan et al., 2013; Duan et al., 2018; Wu et al., 2020). By eliciting employees’ surface compliance, authoritarian leadership exerts a positive indirect effect on employees’ task performance but a negative indirect effect on their workplace well-being (Xiao et al., 2024). In an authoritarian leadership model, the leader monopolizes the decision-making process and expects complete subservience from subordinates. Consequently, subordinates often react with compliance, fearful respect, humiliation, and apprehension (Zheng et al., 2025). Considering that leadership significantly shapes employees’ perceptions and behaviors and is often perceived as a reflection of the organization (Jeong et al., 2023), understanding how authoritarian leadership influences knowledge hiding is crucial. Although empirical study has documented the negative impact of authoritarian leadership on employee well-being in organizations (Dhaneesh et al., 2025), scholarly understanding of the mechanisms linking authoritarian leadership and knowledge hiding remains incomplete. Specifically, we examine how authoritarian leadership, defined as a leadership style characterized by strict control and low tolerance for employee autonomy, shapes employees’ knowledge hiding through the serial multiple mediating roles of hindrance stressors and job insecurity. Drawing on this framework, this study investigates the antecedents of knowledge hiding in the context of Chinese small- and medium-sized firms (SMEs), where hierarchical structures and high-power distance are often salient (Rui et al., 2024).

To explain how authoritarian leadership fosters employees’ knowledge hiding, this study draws on the conservation of resources (COR) theory and the Job Demands Resources (JD-R) model as complementary theoretical foundations. COR theory posits that individuals strive to obtain, retain, and protect valued resources, and that perceived threats to these resources often trigger defensive cognitive and behavioral responses (Hobfoll, 1989). Meanwhile, the JD-R model explains how demanding work environments generate psychological strain when job demands exceed available resources (Bakker and Demerouti, 2007). Integrating these perspectives, we argue that authoritarian leadership functions as a resource threatening workplace condition that intensifies employees’ perceived demands and uncertainty, thereby increasing the likelihood of self protective behaviors such as knowledge hiding. The COR theories suggest that employees’ reactions to leadership are shaped less by leaders’ behaviors per se and more by how employees appraise the demands and threats embedded in those behaviors (Buttigieg et al., 2023). Building on this premise, we propose a sequential psychological pathway through which authoritarian leadership triggers knowledge hiding. Authoritarian leadership is inherently demanding, punitive, and controlling (Chan et al., 2013). Such leaders often impose unnecessary rules, close surveillance, and unclear authority boundaries, thereby amplifying employees’ hindrance stressors (Chen et al., 2024). These stressors accumulate within the tense interpersonal climate often found in authoritarian teams—marked by anger, hostility, and heightened emotional strain—which gradually deteriorates social bonds among members (Karakitapoğlu-Aygün et al., 2023). Consequently, employees view their work environment as high in demand but low in foreseeable rewards, interpreting it as obstructive rather than motivating. Under such conditions, employees’ sense of job insecurity intensifies. When individuals feel constrained by opaque decision-making processes and vulnerable to leaders’ unilateral power, uncertainty about their ongoing roles, status, and future opportunities becomes salient (Tsui et al., 2004). This insecurity depletes emotional and cognitive resources, prompting employees to prioritize self-protection (Li et al., 2024). Knowledge hiding has emerged as a functional, albeit maladaptive coping response. By withholding information, employees attempt to preserve value, avoid potential sanctions, and reduce their exposure to relational or performance risks in hierarchically imbalanced settings (Shaukat et al., 2025). In this study, we consider hindrance stressors and job insecurity together to constitute a serial mediation process explaining how authoritarian leadership fosters knowledge hiding.

This psychological tendency may be further intensified under exposure to hindrance stressors, such as work-related demands, bureaucratic constraints, or role conflict, which are typically appraised as threatening and uncontrollable (Cavanaugh et al., 2000). Job stressors, particularly those characterized as hindrance stressors, deplete psychological resources, thereby fostering emotional exhaustion and disengagement (Cavanaugh et al., 2000). Benítez-Núñez et al. (2024) indicated that hindrance stressors in the workplace are key mechanisms for reducing employee satisfaction and increasing turnover intention. Research on antecedents conceptualizes job insecurity as a multifaceted perception shaped by socio-political conditions and cultural norms at the macro level, organizational and family environments at the meso level, and individual experiences, personality traits, and sociodemographic characteristics at the micro level (Klug et al., 2024). Wang et al. (2024) demonstrate that challenge stressors and hindrance stressors exert significant positive predictive effects on knowledge hiding, and that higher levels of hindrance stressors further increase the likelihood of knowledge hiding behaviors. However, little is known about the underlying psychological mechanisms and contextual pressures through which these stressors act as antecedents.

Another plausible pathway is job insecurity, a chronic psychological unsettling status reflecting employees’ perceived threat of job loss or uncertainty regarding future work continuity (Shoss, 2017). Job insecurity is a primary factor that negatively affects employees’ work-related attitudes, motivation, organizational behavior, and well-being (Cheng and Chan, 2008; Sverke et al., 2019). Furthermore, job insecurity is linked to knowledge hiding, with employees concealing information as a form of resource protection or impression management (Jeong et al., 2023). Supervisors’ authoritarian leadership behavior elicits negative emotions among subordinates, such as anger, hostility, and fear (Farh et al., 2006). This is further supported by Aryee et al. (2007), which indicates a positive correlation between leaders’ autocratic behavior and subordinates’ perceived abusive supervision. Such treatment and the negative emotions it engenders can ultimately lead to a sense of job insecurity among employees (Glambek et al., 2018). Job insecurity, understood as a negative self-cognitive emotion, has been linked to the suppression of innovative behavior and an increase in employee silence (Jiang and Lavaysse, 2018). When basic job security needs are unmet, employees prioritize career concerns over pursuing self-actualization needs such as knowledge sharing. Job insecurity directs employees’ focus toward career concerns, thereby inhibiting their willingness to engage in extra-role behaviors (Yu et al., 2021).

Building upon these theoretical considerations, this study makes three key contributions:

First, it extends authoritarian leadership research by examining its influence on knowledge hiding, an outcome of growing relevance that remains insufficiently addressed in the existing literature. Existing studies on authoritarian leadership have primarily concentrated on its detrimental consequences for employees within organizational settings, such as reduced service performance (Thawornlamlert et al., 2026), diminished work engagement (Dhaneesh et al., 2025), and lower levels of employee voice (Liu and Ling, 2025). While the extant literature confirms a general negative relationship between authoritarian leadership and pro-social behaviors (Yang et al., 2025), it often overlooks the precise psychological pathway through which this dominance and control compel employees to engage in secretive behaviors, such as knowledge hiding. Second, drawing on the Job Demands Resources (JD-R) framework and the conservation of resources (COR) theory (Hobfoll, 1989), this study introduces hindrance stressors and job insecurity as serial mediators to explain how authoritarian leadership fosters knowledge hiding. By testing this sequential psychological mechanism, the study addresses the limited empirical understanding of the underlying process linking authoritarian leadership to knowledge hiding. Nevertheless, it answers recent calls for more integrative research on how workplace stressors and power asymmetries jointly influence counterproductive knowledge behaviors (Afshar-Jalili et al., 2021; Tang et al., 2022). Third, by situating the model within Chinese SMEs, this study addresses an important contextual gap in the literature on leadership and knowledge behaviors. The cultural context, marked by hierarchy, relational dependence, and a preference for stability, may also increase employee sensitivity to leader pressure and job-related threats (Probst and Lawler, 2006). Chinese SMEs, marked by uncertain environments, concentrated authority, and limited opportunities for advancement, provide a meaningful setting for examining this process (Parnell et al., 2015). Insights from this setting can help Chinese SMEs overcome knowledge barriers that restrict their long-term development. In summary, this study developed and empirically tested a serial mediation framework in which authoritarian leadership increases employees’ hindrance stressors, which subsequently heighten job insecurity and ultimately lead to knowledge hiding.

## 2 Theoretical background: hindrance stressors, job insecurity, and knowledge hiding

### 2.1 Authoritarian leadership and knowledge hiding

Authoritarian leaders reinforce their personal dominance over subordinates by leveraging power asymmetry, centralizing authority, and exercising control through unilateral decision-making (Tsui et al., 2004). In such environments, subordinates are often discouraged from expressing opinions or deviating from the prescribed norms, resulting in constrained psychological agency and increased perceived vulnerability (Harms et al., 2017). Authoritarian leaders frequently overlook employees’ needs and viewpoints, creating unmet expectations that erode organizational integrity, heighten cynicism, and elicit dehumanized responses, ultimately intensifying employee burnout (Jiang et al., 2019). Emotional exhaustion reflects the depletion of essential resources and increases employees’ sensitivity to potential threats (Prapanjaroensin et al., 2017). Under the lens of the COR theory (Hobfoll, 1989), managers’ emotional exhaustion signals a resource-depleted environment, prompting employees to anticipate a heightened risk of resource loss. Faced with this threat, employees activate defensive resource-preservation motives and increasingly view knowledge-sharing as a costly behavior that consumes cognitive and emotional resources. Consequently, they are inclined to engage in knowledge hiding to retain their resources and protect themselves from further depletion. Unlike mere silence or a lack of sharing, knowledge hiding is an active behavior rooted in motives, often triggered by perceived threats to status, autonomy, or survival within a hierarchical structure (Peng, 2013).

From the perspective of the COR theory (Hobfoll, 1989), employees are motivated to retain and protect valued resources when facing threatening or resource depleting workplace conditions. Authoritarian leadership, characterized by strict control, excessive monitoring, and unquestioning obedience, may heighten employees’ perceptions of resource loss, including diminished autonomy, reduced psychological safety, and increased uncertainty. Under such conditions, employees are more likely to adopt defensive strategies to preserve personal value and reduce replaceability. In hierarchical environments where knowledge is closely tied to power, status, and personal security, employees may withhold knowledge as a self protective response (Yuan et al., 2021). Based on this reasoning, we propose the following hypotheses:

*Hypothesis 1a*: Authoritarian leadership will have a positive impact on playing dumb.

*Hypothesis 1b*: Authoritarian leadership will have a positive impact on evasive hiding.

*Hypothesis 1c*: Authoritarian leadership will have a positive impact on rationalized hiding.

*Hypothesis 1d*: Authoritarian leadership will have a positive impact on procrastination.

### 2.2 The mediating effect of hindrance stressors

The JD-R model posits that the work environment consists of two fundamental elements: job demands and job resources. High job demands drain employees’ energetic, emotional, and cognitive resources, while sufficient job resources such as autonomy, psychological safety, and social support help replenish them and sustain motivation (Bakker and Demerouti, 2007; Demerouti et al., 2001). In case of high demands and low resources, employees face an energetic depletion process that often leads to defensive rather than proactive resource-protecting behaviors. Within the JD-R framework, authoritarian leadership can be understood as a contextual force that increases job demands and erodes critical job resources. Authoritarian leadership is characterized by unilateral control, punitive supervision, strict monitoring, and unpredictable standards that raise administrative constraints, emotional strain, role ambiguity, and compliance pressure (Chan et al., 2013). These conditions correspond to hindrance stressors, which are a subset of job demands that block goal attainment and deplete resources. Authoritarian leadership also undermines autonomy, perceived support, and psychological safety—key job resources that employees rely on to cope with their demands (Aryee et al., 2007). Thus, authoritarian leadership intensifies demand, while simultaneously shrinking the resource pool.

From the perspective of the JD-R framework, the combination of hindrance stressors and limited resources signals a demanding, uncontrollable work environment with little return on investment. Consequently, employees are motivated to conserve their cognitive and emotional resources. Defensive coping strategies replace proactive behaviors. Within this resource-conservation cycle, the intentional withholding of knowledge (i.e., knowledge hiding) becomes a particularly functional response. Knowledge hiding is defined as a deliberate attempt to withhold or conceal knowledge requested by a person. It is a distinct construct involving an active decision to deny or obscure requested information, rather than simply the absence of sharing (Connelly et al., 2012). Hindrance stressors reflect job demands that obstruct goal attainment and are perceived as uncontrollable, resource-depleting constraints rather than motivating challenges. Consequently, employees under authoritarian leadership are more likely to experience feelings of entrapment, incapability, and threat, and to interpret leader behaviors as obstacles that hinder effective performance. When employees confront hindrance stressors, the resulting psychological strain leads them to conserve their limited time and energy by avoiding discretionary activities beyond their formal roles, thereby reducing engagement in organizational citizenship behavior (Pooja et al., 2016). Although knowledge hiding comprises multiple behavioral manifestations, including playing dumb, evasive hiding, rationalized hiding, and procrastination, the present study theorizes knowledge hiding primarily as a resource preservation response to authoritarian leadership and workplace threats. The multidimensional operationalization is adopted to capture behavioral heterogeneity and examine the robustness of the proposed relationships across manifestations. Based on this reasoning, we propose the following hypothesis:

*Hypothesis 2a*: Hindrance stressors will positively mediate the relationship between authoritarian leadership and playing dumb.

*Hypothesis 2b*: Hindrance stressors will positively mediate the relationship between authoritarian leadership and evasive hiding.

*Hypothesis 2c*: Hindrance stressors will positively mediate the relationship between authoritarian leadership and rationalized hiding.

*Hypothesis 2d*: Hindrance stressors will positively mediate the relationship between authoritarian leadership and procrastination.

### 2.3 The mediating effect of job insecurity

According to the COR theory (Hobfoll, 1989), individuals are motivated to retain and protect valued resources such as employment continuity, reputation, and role security. Authoritarian leadership is characterized by five awe-inspiring (li-wei) behaviors—powerfully subduing, authority and control, intention hiding, rigor, and doctrinism—that commonly elicit obedience, compliance, and fear among subordinates. In addition, this type of leadership asserts absolute authority and control over subordinates and demands their unquestioning obedience, which is best conceptualized not as a static condition but as a dynamic and anticipatory appraisal involving cognitive evaluations of potential job loss and affective responses to perceived threats—processes often intensified by ambiguous or controlling leadership behaviors (Jiang and Lavaysse, 2018). When facing authoritarian leadership, employees may perceive a threat to their core resources such as status (Duan et al., 2018), autonomy (Pizzolitto et al., 2023), and psychological safety (David and Shih, 2025), triggering worry and fear of job continuity (Vander Elst et al., 2014). Once job insecurity is triggered, employees shift to defensive behavioral modes, as explained by the COR theory (Hobfoll, 1989). In this state, individuals aim to protect their remaining tangible and intangible resources. Therefore, employees may engage in knowledge hiding as a self-protective response to minimize replaceability, retain perceived uniqueness, and maintain political leverage rather than sharing information to enhance collective performance (Peng, 2013; Serenko and Bontis, 2016).

Authoritarian leadership is a leadership style in which leaders assert absolute authority and demand unquestioning obedience, often using personal discretion and coercive tactics to control and motivate subordinates. Repeated dominance by authoritarian leaders can cultivate an organizational environment characterized by control and compliance, potentially leading to abusive or toxic leadership styles, especially in high-stress settings (Harms et al., 2017). Such leadership that demands unquestioning obedience and enforces compliance through punitive measures can ensure task completion; however, it often imposes psychological costs on employees (Chan et al., 2013). One such psychological cost is job insecurity, defined as the fear of losing the present job due to unexpected and uncontrollable reasons that threaten the continuity of work experience (Shoss, 2017). Job insecurity is influenced not only by macroeconomic factors but also by leadership behaviors (Probst, 2005). By exercising rigid power and control, authoritarian leaders can instill the fear of sanctions, favoritism, or arbitrary dismissal in subordinates, thereby amplifying their sense of insecurity (Schilling, 2009). Moreover, authoritarian leadership weakens employees’ organizational self-esteem by limiting participation and recognition, making them feel less valued and dispensable (Duan et al., 2018)—conditions which naturally lead to job insecurity (Kinnunen et al., 2003).

Job insecurity, as a subjective appraisal of threats to job continuity or valued employment conditions, reflects employees’ anticipation of adverse employment outcomes that may compromise their stability, predictability, and organizational embeddedness (Greenhalgh and Rosenblatt, 1984; Jiang and Lavaysse, 2018). Individuals experiencing job insecurity are likely to engage in self-protective behaviors, often at the expense of collective goals (Probst, 2005). One such behavior is knowledge hiding, which refers to the intentional concealment of knowledge requested by others (Connelly et al., 2012).

The mediating effect of job insecurity is theoretically grounded in the COR theory (Hobfoll, 1989), which suggests that perceived threats to key resources, including position, influence, and expertise, prompt individuals to act defensively to conserve the remaining resources. As a valuable intangible asset, knowledge may be withheld as a strategic means of enhancing perceived indispensability and protecting one’s competitive edge within an organization (Serenko and Bontis, 2016). In such contexts, knowledge hiding may serve as a protective strategy to reduce perceived replaceability, help insecure employees safeguard their values, and mitigate feelings of vulnerability. In authoritarian contexts, the depletion of psychological and emotional resources may prompt employees to engage in knowledge hiding as a self-protective response to avoid further strain and maintain control (Asim et al., 2021). Additionally, the micropolitical dynamics of such environments may lead employees to view knowledge as a form of private capital, strategically withhold it to preserve its indispensability, and navigate perceived organizational instability (Evans et al., 2015).

Therefore, we propose the following hypothesis:

*Hypothesis 3a*: Job Insecurity will positively mediate the relationship between authoritarian leadership and playing dumb.

*Hypothesis 3b*: Job Insecurity will positively mediate the relationship between authoritarian leadership and evasive hiding.

*Hypothesis 3c*: Job Insecurity will positively mediate the relationship between authoritarian leadership and rationalized hiding.

*Hypothesis 3d*: Job Insecurity will positively mediate the relationship between authoritarian leadership and procrastination.

### 2.4 The serial mediating effect of hindrance stressors and job insecurity

Authoritarian leadership, characterized by unilateral control, strict monitoring, and punitive enforcement, signals low autonomy and suppresses subordinates’ voices (Chan et al., 2013). Such leadership practices increase administrative constraints and interpersonal tension, acting as contextual antecedents of hindrance stressors. These stressors deplete employees’ psychological resources and generate sustained strain (LePine et al., 2005). Such resource draining effects may become particularly salient in authoritarian contexts, where controlling supervision imposes additional demands while limiting employees’ opportunities to resolve or reframe work related stressors (Feng et al., 2024). The gradual accumulation of hindrance stressors undermines employees’ sense of psychological safety and personal agency, increasing their perception of job insecurity. When the work environment combines structural impediments with authoritarian control, employees interpret leadership behaviors as coercive threats to their role stability and prospects (Keltner et al., 2003; Haugaard, 2025). This compounded exposure—external control from leaders and internal organizational constraints—produces a power–threat dynamic in which employees feel more vulnerable and uncertain about employment continuity (Keltner et al., 2003). Consistent with the COR theory, such perceived insecurity reflects further resource depletion and motivates defensive strategies aimed at self-preservation (Hobfoll, 1989). Knowledge hiding is a prominent defensive response against heightened insecurity. By withholding or delaying knowledge, employees seek to protect their value, reduce exposure to punitive scrutiny, and preserve scarce cognitive and emotional resources (Bogilović et al., 2017). In particular, employees are more likely to engage in evasive hiding, playing dumb, rationalized hiding, or procrastination-style hiding as tactical means to conserve resources and manage threats when hindrance stressors are salient and job insecurity increases. Thus, hindrance stressors mediate the effect of authoritarian leadership on job insecurity and initiate a resource conservation cascade that culminates in knowledge hiding.

This association can be further explained by the COR theory (Hobfoll, 1989), which posits that individuals strive to acquire and preserve valuable resources, including job stability. Authoritarian leadership can shape subordinates’ perceptions of abusive supervision, as such leaders assert personal dominance and rely on threats and intimidation to regulate their behavior, thereby increasing the likelihood of being perceived as abusive (Kiazad et al., 2010). Such perceptions of supervisory mistreatment trigger employees’ hindrance stressors (Li et al., 2022), which represent work conditions that obstruct personal growth and goal attainment, thereby placing employees in a state of sustained strain (Dawson et al., 2016). When employees face persistent role ambiguity, bureaucratic constraints, or organizational politics, they struggle to form clear expectations about their future work outcomes and positional stability. Over time, this ambiguity diminishes their sense of predictability and control–two essential psychological resources that help them maintain confidence in the continuity of their employment (Junça Silva and Rodrigues, 2024). As these stressors accumulate, employees become increasingly concerned about negative evaluations or unfavorable organizational decisions, leading them to question their job security (Rangrez et al., 2022). Rather than merely withholding discretionary behavior, employees may increasingly engage in knowledge hiding as job insecurity intensifies, using deliberate knowledge concealment to protect their interests and maintain a sense of security under perceived employment threats (Černe et al., 2014). Empirical research supports this relationship, showing that higher levels of JI are associated with stronger tendencies to engage in defensive knowledge behaviors, particularly when competition or resource scarcity is salient (Jeong et al., 2022).

Based on the above theoretical background, we propose the following hypotheses:

*Hypothesis 4a*: Hindrance stressors and job insecurity will have a positive serial multiple mediating effect between authoritarian leadership and playing dumb.

*Hypothesis 4b*: Hindrance stressors and job insecurity will have a positive serial multiple mediating effect between authoritarian leadership and evasive hiding.

*Hypothesis 4c*: Hindrance stressors and job insecurity will have a positive serial multiple mediating effect between authoritarian leadership and rationalized hiding.

*Hypothesis 4d*: Hindrance stressors and job insecurity will have a positive serial multiple mediating effect between authoritarian leadership and procrastination.

## 3 Methods

### 3.1 Sample and procedure

To examine the effect of authoritarian leadership on knowledge hiding, we surveyed organizational members working in Chinese SMEs using an online questionnaire. Managers of SMEs were first contacted to distribute the survey link to eligible employees. To enhance participation, respondents were recruited through social media platforms such as WeChat. A total of 363 data samples were collected and used for empirical analysis.

The sample consisted of employees aged 18 years and older who were currently employed in SMEs. The survey clearly described the purpose of the study, assured participants of anonymity, emphasized the voluntary nature of participation, and confirmed that neither participants nor their organizations would incur any adverse consequences for participating in the study.

For data analysis, SPSS version 26.0 was first employed to conduct demographic analysis, exploratory factor analysis, reliability assessment, descriptive statistics, correlation analysis, and tests of moderation hypotheses. AMOS 23.0 was subsequently used to perform confirmatory factor analysis (CFA), discriminant validity assessment, and path analysis. Finally, the moderated mediation hypothesis was examined using SPSS PROCESS Macro 3.4.1.

Regarding the participants’ demographic characteristics, 172 (47.4%) were male and 191 (52.6%) females. Two (0.6%) participants were aged under 20 years, 148 (40.8%) were aged 20–30 years, 84 (23.1%) were aged 31–40 years, 68 (18.7%) were aged 41–50 years, 57 (15.7%) were 51–60 years, and 4 (1.1%) were over 60 years old. Regarding education, 118 participants (32.5%) had an associate’s degree or lower, 149 (41.0%) had a bachelor’s degree, 72 (19.8%) had a master’s degree, and 24 (6.6%) had a doctoral degree. In terms of years of service, 60 (16.5%) participants had worked for less than 1 year, 68 (18.7%) for one to less than 3 years, 33 (9.1%) for three to less than 5 years, 34 (9.4%) for five to less than 7 years, and 168 (46.3%) for 7 years or more.

Figure 1 presents the proposed research model, which examines the relationships among authoritarian leadership, hindrance stressors, job insecurity, and knowledge hiding. The model proposes that authoritarian leadership influences knowledge hiding both directly and indirectly through hindrance stressors and job insecurity. Knowledge hiding is conceptualized as a multidimensional construct comprising playing dumb, evasive hiding, rationalized hiding, and hiding procrastination.

**Figure 1:**
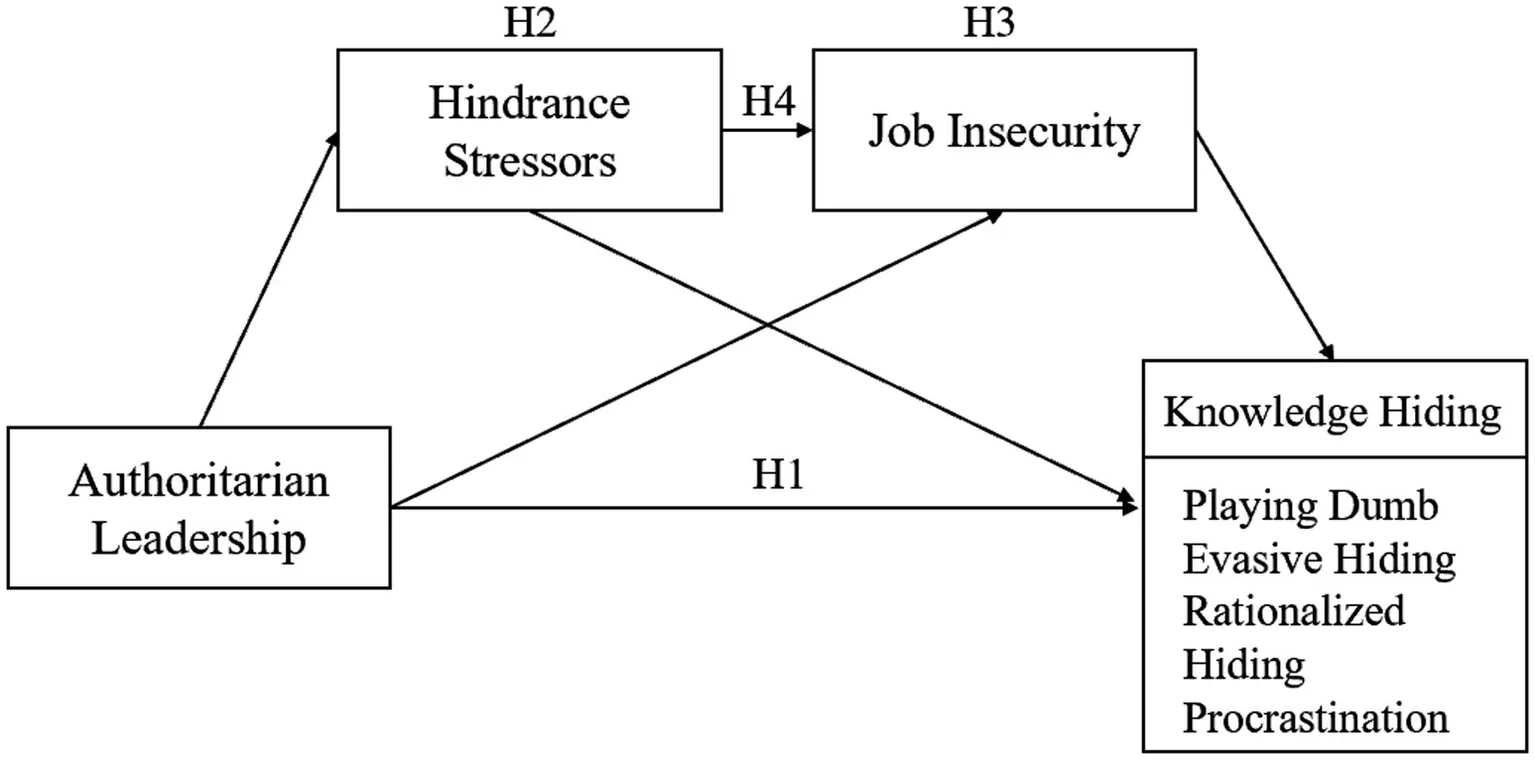
Research model.

### 3.2 Measurement

#### 3.2.1 Authoritarian leadership

Authoritarian leadership is operationalized as employees’ perception of a leadership style characterized by the exertion of absolute authority, the expectation of unquestioning obedience, and the use of personal discretion and threats to control and influence subordinates’ behavior. The authoritarian leadership scale developed by Zheng et al. (2006) has been widely applied and empirically validated in the Chinese context (Li et al., 2012), which captures subordinates’ perceptions of their leader’s authoritarian behaviors. Sample items include: “My leader always behaves in a commanding fashion in front of employees,” and “We have to follow the leader’s rules to get things done. Otherwise, they punish us severely.”

#### 3.2.2 Job insecurity

Job insecurity is a subjective perception of threats to the continuity and stability of employment, reflecting an overall sense of uncertainty and concern about the future of one’s job (Greenhalgh and Rosenblatt, 1984). We measured this construct by using the Job Insecurity Scale by Yam et al. (2023), which captures employees’ cognitive evaluations of potential job losses. Sample items include “I feel insecure about the future of my job” and “I think I might lose my job in the near future.”

#### 3.2.3 Knowledge hiding

Knowledge hiding is an intentional attempt by an individual to withhold or conceal knowledge requested by another person. It reflects a form of self-protective or strategic behavior aimed at preserving informational advantages or avoiding perceived threats in interpersonal and organizational contexts (Jin et al., 2023). We assessed this construct by using the scale developed and validated in their study, which captures multiple dimensions of knowledge-hiding behavior. Sample items include: “I pretended that I did not know the information” and “I agreed to help my colleague but never really intended to.”

#### 3.2.4 Hindrance stressors

Hindrance stressors, including organizational politics, red tape, role ambiguity, and concerns about job security, are perceived as unnecessary obstacles to personal growth and goal attainment (LePine et al., 2005). We measured this construct by using the scale by LePine et al. (2016), which captures the degree to which employees view their jobs as impeding their personal accomplishments. Sample items include: “Working to fulfill the demands of my job thwarts my personal growth and well-being,” and “I feel the demands of my job constrain my achievement of personal goals and development.”

All items were measured using a 7-point Likert scale ranging from 1 (strongly disagree) to 7 (strongly agree). Figure 1 illustrates the research model used in this study.

### 3.3 Statistical analysis

The statistical procedures used in this study were conducted in several stages. We first performed a demographic analysis, followed by CFA. We then assessed the reliability of all the measurement instruments and conducted a correlation analysis to examine the associations among the variables. Regression analyses were used to test the hypothesized relationships. All demographic, reliability, descriptive, correlation, and regression analyses were conducted using the SPSS 26.0. CFA and path analysis were performed with AMOS 23.0. Finally, the moderated mediation model was tested using PROCESS Macro 3.4.1 (Model 6).

## 4 Results

### 4.1 Confirmatory factor and reliability analyses

CFA confirmed the applicability of the data and different models (Bandura and Schunk, 1981), and structural equation modeling was performed using AMOS 22.0. The four-factor model displayed good fit, reliability, and validity. Next, the convergent validity was tested, and the results showed that the standardized regression weights of authoritarian leadership, hindrance stressors, job insecurity, and job performance ranged from 0.764 to 0.899, 0.919 to 0.939, 0.898 to 0.929, and 0.880 to 0.942, respectively. We also analyzed the average variance extracted (AVE) and composite reliability (CR). The AVE values for authoritarian leadership, hindrance stressors, job insecurity, playing dumb, evasive hiding, rationalized hiding and procrastination were 0.707, 0.864, 0.834, 0.867, 0.874, 0.792, and 0.800, respectively. These results show that all AVE values were greater than 0.5. The CR values for authoritarian leadership, hindrance stressors, job insecurity, playing dumb, evasive hiding, rationalized hiding and procrastination were 0.833, 0.750, 0.750, 0.800, 0.800, 0.800, and 0.916, respectively. Thus, these results show that all CR values were higher than 0.7.

Furthermore, we tested the model fit index, which included the absolute fit, incremental fit, and parsimonious adjusted indices. The results of the model fit index showed *χ*^2^(*p*) = 833.417 (0.000), *χ*^2^/df = 1.664, and GFI = 0.882, RMSEA = 0.043. This index is considered a good fit if it is less than or equal to 3, and a fair fit if it is less than or equal to 5 (Hong, 2000). RMSEA is considered a good fit if it is 0.05 or less, fair fit if it is 0.08, moderate fit if it is less than 0.10, and poor fit if it is greater than 0.10 (Browne and Cudeck, 1992). The incremental fit indices are as follows: IFI = 0.980, NFI = 0.951, TLI = 0.978, RFI = 0.946, and CFI = 0.980. If this index is greater than 3 and is 0.9, it is considered a good fit (Wang and Wang, 2019). The parsimony-adjusted indices were PNFI = 0.850, AGFI = 0.859, AIC = 1021.417, and PGFI = 0.742. This index is considered to be between 0 and 1 (with 0 or 1 outside this range). The results are summarized in Table 1.

**Table 1:** Results of confirmatory factor and reliability analyses.

Variables		Estimate	S.E.	C.R.	*p*	Standardized regression weights	Average variance extracted (AVE)	Composite reliability (CR)
Authoritarian leadership (A)	A1	1				0.764	0.707	0.833
	A2	1.021	0.052	19.626	***	0.774		
	A3	1.218	0.067	18.208	***	0.889		
	A4	1.188	0.064	18.454	***	0.899		
	A5	1.209	0.068	17.719	***	0.868		
Hindrance stressor (B)	B1	1				0.919	0.864	0.750
	B2	0.999	0.032	30.906	***	0.93		
	B3	1.085	0.034	31.712	***	0.939		
Job insecurity (C)	C1	1				0.912	0.834	0.750
	C2	0.95	0.036	26.762	***	0.898		
	C3	0.998	0.034	28.94	***	0.929		
Playing dumb (D)	D1	1				0.926	0.867	0.800
	D2	1.029	0.03	34.316	***	0.94		
	D3	1.035	0.03	34.492	***	0.941		
	D4	0.999	0.032	31.452	***	0.917		
Evasive hiding (E)	E1	1				0.936	0.874	0.800
	E2	0.967	0.029	33.797	***	0.926		
	E3	1.015	0.028	36.623	***	0.944		
	E4	0.971	0.028	35.015	***	0.934		
Rationalized hiding (F)	F1	1				0.931	0.792	0.800
	F2	0.912	0.036	25.63	***	0.861		
	F3	0.942	0.037	25.51	***	0.859		
	F4	0.965	0.04	24.417	***	0.907		
Procrastination (G)	G1	1				0.917	0.800	0.916
	G2	1.007	0.028	35.727	***	0.926		
	G3	1.053	0.031	33.537	***	0.942		
	G4	0.981	0.035	28.288	***	0.892		
	G5	0.969	0.033	29.6	***	0.906		
	G6	0.92	0.033	27.577	***	0.884		
	G7	1	0.032	31.584	***	0.925		
	G8	1.019	0.032	31.627	***	0.926		
	G9	0.668	0.04	16.497	***	0.687		
	G10	0.957	0.034	28.401	***	0.894		
	G11	0.978	0.032	30.437	***	0.915		

*χ*^2^(*p*) = 833.417, *p* < 0.001, *χ*^2^/df = 1.664, GFI = 0.882, NFI = 0.951, RFI = 0.946, IFI = 0.980, TLI = 0.978, CFI = 0.980, RMSEA = 0.043, PNFI = 0.850, AGFI = 0.859, AIC = 1021.417, PGFI = 0.742.

***p < 0.001.

### 4.2 Descriptive statistics and correlation analysis

Descriptive statistics were calculated for all study variables, including mean and standard deviation. The mean scores for authoritarian leadership, hindrance stressors, job insecurity, playing dumb, evasive hiding, rationalized hiding, and procrastination were 4.511, 4.221, 4.277, 4.032, 3.968, 4.306, and 4.109, respectively, and the corresponding standard deviations were 1.618, 1.732, 1.808, 1.845, 1.923, 1.736, and 1.716, respectively. Following Cohen (1988), correlation coefficients of approximately 0.10, 0.30, and 0.50 were interpreted as weak, moderate, and strong relationships, respectively. The results showed that authoritarian leadership was moderately and positively correlated with hindrance stressors (*r* = 0.491, *p* < 0.001), job insecurity (*r* = 0.429, *p* < 0.001), playing dumb (*r* = 0.429, *p* < 0.001), evasive hiding (*r* = 0.425, *p* < 0.001), rationalized hiding (*r* = 0.407, *p* < 0.001), and procrastination (*r* = 0.424, *p* < 0.001). Hindrance stressors showed strong positive correlations with job insecurity (*r* = 0.558, *p* < 0.001), playing dumb (*r* = 0.556, *p* < 0.001), evasive hiding (*r* = 0.584, *p* < 0.001), and procrastination (*r* = 0.564, *p* < 0.001), while its correlation with rationalized hiding was moderate (*r* = 0.499, *p* < 0.001). Job insecurity was strongly and positively correlated with playing dumb (*r* = 0.567, *p* < 0.001), evasive hiding (*r* = 0.591, *p* < 0.001), and procrastination (*r* = 0.557, *p* < 0.001), while its correlation with rationalized hiding was moderate (*r* = 0.496, *p* < 0.001). Strong positive correlations were also observed among the four dimensions of knowledge hiding, with coefficients ranging from 0.850 to 0.939 (all *p* < 0.001). Descriptive statistics and correlations are presented in Table 2.

**Table 2:** Results of descriptive statistics and correlation analysis.

Variables	Mean	Standard deviation	A	B	C	D	E	F	G
Authoritarian leadership (A)	4.511	1.618	0.707						
Hindrance stressors (B)	4.221	1.732	0.491*** (0.241)	0.864					
Job insecurity (C)	4.277	1.808	0.429***	0.558*** (0.311)	0.834				
Playing dumb (D)	4.032	1.845	0.429***	0.556***	0.567*** (0.321)	0.867			
Evasive hiding (E)	3.968	1.923	0.425***	0.584***	0.591***	0.931*** (0.867)	0.874		
Rationalized hiding (F)	4.306	1.736	0.407***	0.499***	0.496***	0.850***	0.858*** (0.736)	0.792	
Proscrastination (G)	4.109	1.716	0.424***	0.564***	0.557***	0.934***	0.939***	0.887*** (0.787)	0.800

***p < 0.001.

To assess the discriminant validity, the maximum squared correlation among the variables was compared with each variable’s AVE. This comparison followed the standard criterion that the AVE should exceed the highest shared variance. In Table 2, the values shown in parentheses after each correlation coefficient represent the square roots of the variables’ maximum squared correlations, which facilitates comparison with the correlations. AVE values were also added to the table to allow a direct evaluation of discriminant validity. Overall, all statistical results aligned with expectations, the relationships among variables were significant in the anticipated directions, and discriminant validity was clearly established.

Following Kock (2015), full collinearity was assessed using variance inflation factors (VIFs). The VIF values for authoritarian leadership (1.381), hindrance stressors (1.635), and job insecurity (1.522) were all below the recommended threshold of 3.3, suggesting that common method bias was unlikely to be a serious concern.

### 4.3 Hypothesis test

This study applied Model 6 of SPSS PROCESS Macro version 3.4. to test the hypotheses. The results demonstrated that authoritarian leadership positively influenced hindrance stressors (estimate = 0.525, *p* < 0.001), job insecurity (estimate = 0.229, *p* < 0.001), playing dumb (estimate = 0.155, *p* < 0.01), evasive hiding (estimate = 0.129, *p* < 0.05), rationalized hiding (estimate = 0.167, *p* < 0.01), and procrastination (estimate = 0.136, *p* < 0.01). Hindrance stressors had positive effects on job insecurity (estimate = 0.477, *p* < 0.001), playing dumb (estimate = 0.317, *p* < 0.001), evasive hiding (estimate = 0.366, *p* < 0.001), rationalized hiding (estimate = 0.267, *p* < 0.001), and procrastination (estimate = 0.319, *p* < 0.001). In addition, job insecurity had positive effects on playing dumb (estimate = 0.350, *p* < 0.001), evasive hiding (estimate = 0.384, *p* < 0.001), rationalized hiding (estimate = 0.269, *p* < 0.001), and procrastination (estimate = 0.306, *p* < 0.001).

Hypothesis 1 assessed the direct effects of authoritarian leadership on playing dumb, evasive hiding, rationalized hiding, and procrastination. The positive effects were 0.155 [0.049, 0.261], 0.129 [0.022, 0.236], 0.167 [0.060, 0.273], and 0.136 [0.037, 0.235]. As zero is absent from the interval, the serial mediation effect is significant, supporting Hypothesis 4. The detailed indirect effects along with their LLCI and ULCI values are reported in Table 3.

**Table 3:** Results of serial multiple mediation.

Path	Estimate	SE	t	*p*	LLCI	ULCI
Authoritarian leadership → hindrance stressors	0.525	0.049	10.703	0.000	0.429	0.622
Authoritarian leadership → job insecurity	0.229	0.055	4.173	0.000	0.121	0.336
Authoritarian leadership → playing dumb	0.155	0.054	2.869	0.004	0.049	0.261
Authoritarian leadership → evasive hiding	0.129	0.055	2.362	0.019	0.022	0.236
Authoritarian leadership → rationalized hiding	0.167	0.054	3.070	0.002	0.060	0.273
Authoritarian leadership → proscrastination	0.136	0.050	2.710	0.007	0.037	0.235
Hindrance stressors → job insecurity	0.477	0.051	9.323	0.000	0.377	0.578
Hindrance stressors → playing dumb	0.317	0.055	5.777	0.000	0.209	0.425
Hindrance stressors → evasive Hiding	0.366	0.055	6.592	0.000	0.257	0.475
Hindrance stressors → rationalized hiding	0.267	0.055	4.850	0.000	0.159	0.376
Hindrance stressors → proscrastination	0.319	0.051	6.229	0.000	0.218	0.419
Job insecurity → playing dumb	0.350	0.051	6.916	0.000	0.251	0.450
Job insecurity → evasive hiding	0.384	0.051	7.486	0.000	0.283	0.484
Job insecurity → rationalized hiding	0.269	0.051	5.285	0.000	0.169	0.370
Job insecurity → proscrastination	0.306	0.047	6.469	0.000	0.213	0.399

Indirect effect of X on Y	Estimate	SE	LLCI	ULCI
Authoritarian leadership → hindrance stressors → playing dumb	0.166	0.038	0.096	0.245
Authoritarian leadership → hindrance stressors → evasive hiding	0.192	0.041	0.118	0.276
Authoritarian leadership → hindrance stressors → rationalized hiding	0.141	0.038	0.071	0.219
Authoritarian leadership → hindrance stressors → proscrastination	0.167	0.036	0.101	0.243
Authoritarian leadership → job insecurity → playing dumb	0.080	0.027	0.032	0.140
Authoritarian leadership → job insecurity → evasive hiding	0.074	0.024	0.030	0.123
Authoritarian leadership → job insecurity → rationalized hiding	0.062	0.022	0.023	0.110
Authoritarian leadership → job insecurity → proscrastination	0.070	0.025	0.027	0.123
Authoritarian leadership → hindrance stressors→ job insecurity → playing dumb	0.077	0.019	0.044	0.119
Authoritarian leadership → hindrance stressors→ job insecurity → evasive hiding	0.081	0.019	0.048	0.120
Authoritarian leadership → hindrance stressors → job insecurity→ rationalized hiding	0.068	0.019	0.033	0.110
Authoritarian leadership → hindrance stressors → job insecurity→ proscrastination	0.077	0.019	0.043	0.119

Hypothesis 2 examines the mediating role of hindrance stressors in the relationship between authoritarian leadership and the four dimensions of knowledge hiding. As all indirect effects were positive and their confidence intervals excluded zero, the mediation effects were significant, supporting Hypotheses 1a, 1b, 1c, and 1d. The detailed indirect effects along with their LLCI and ULCI values are reported in Table 3.

Hypothesis 3 tested the mediating role of job insecurity on the relationship between authoritarian leadership and the four dimensions of knowledge hiding. As all indirect effects were positive and their confidence intervals excluded zero, the mediation effects were significant, supporting Hypotheses 2a, 2b, 2c, and 2d. The detailed indirect effects along with their LLCI and ULCI values are reported in Table 3.

Hypothesis 4 assessed the serial multiple mediation effects of hindrance stressors and four dimensions of knowledge hiding. The serial indirect effect was 0.1723 with LLCI = 0.0690 and ULCI = 0.3028. As zero is absent from the interval, the serial mediation effect is significant, supporting Hypothesis 3. The detailed indirect effects along with their LLCI and ULCI values are reported in Table 3.

The results of the path analysis are presented in Table 3.

## 5 Discussion

### 5.1 Theoretical implications

This study offers several important theoretical implications for knowledge hiding and leadership.

First, prior research has acknowledged various factors that trigger knowledge hiding, such as supervisor incivility and work disengagement. However, limited attention has been paid to the specific leadership mechanisms that shape this behavior. Drawing on the COR theory, social exchange theory, and the JD-R model, our findings clarify how authoritarian leadership creates conditions that increase employees’ knowledge hiding. The characteristics of authoritarian leadership, such as unilateral control, suppressed employee voice, and punitive supervisory behaviors, create a work environment characterized by unpredictability, restricted autonomy, and low interpersonal respect (Chan et al., 2013). By delineating the links between authoritarian leadership and knowledge hiding, this study contributes to a more nuanced understanding of the hidden costs associated with authoritarian control. These findings enrich the leadership scholarship by extending theoretical discussions on the detrimental impacts of authoritarian authority, thus offering a more comprehensive framework. Although this research has substantially advanced our understanding of how authoritarian control undermines employee outcomes, it remains incomplete in one important respect. Far less attention has been devoted to the implications of authoritarian leadership for knowledge management processes, an area central to organizational learning and strategic renewal. To address this gap, the present study aimed to clarify how authoritarian leadership shapes employees’ work behaviors, with particular emphasis on the mechanism that leads to knowledge hiding. Furthermore, this study provides evidence-based insights highlighting why authoritarian leadership may undermine organizational knowledge flows and inhibit positive employee behaviors. These insights offer new theoretical and practical considerations for designing leadership development initiatives, especially in contexts where healthy interpersonal dynamics and open communication are crucial for long-term organizational functioning.

Second, this study expands the existing literature by advancing the multidimensional understanding of knowledge hiding. Unlike prior research, which has predominantly treated knowledge hiding as a unidimensional construct, this study draws on the framework proposed by Jin et al. (2023) to investigate knowledge hiding across four distinct dimensions: playing dumb, evasive hiding, rationalized hiding, and procrastination. The empirical results demonstrated that authoritarian leadership, hindrance stressors, and job insecurity had significant positive effects on each of the four dimensions. These findings enrich theoretical conversation by showing that different forms of knowledge withholding share common psychological and contextual antecedents, even though they manifest through varied behavioral expressions. This study offers a more nuanced understanding of how negative leadership and stressful work environments shape employees’ selective withholding strategies. It also broadens the conceptual boundaries of knowledge hiding research and provides a valuable reference point for future studies on its heterogeneity.

Third, this study provides empirical evidence for a sequential mediating mechanism through which hindrance stressors and job insecurity transmit the effects of authoritarian leadership on knowledge hiding. By uncovering this psychological transmission process, the present study extends knowledge hiding research through the integration of structural leadership styles and psychological strain as critical antecedents, while also advancing the understanding of authoritarian leadership by revealing how its influence unfolds through employees’ perceptions of hindrance stressors and job insecurity. Authoritarian leadership suppresses employees’ willingness to speak, prompting them to remain silent and withhold suggestions or feedback. Such employee silence not only weakens the flow of information within the organization but also hinders the employees’ sense of involvement and ownership (Duan et al., 2018). This dynamic arises because authoritarian leaders often convey threatening signals through criticism, commands, and displays of authority, leading employees to perceive high risks associated with voicing their opinions, thereby diminishing their job insecurity. Perceived insecurity, in turn, reduces subordinates’ willingness to share information, thereby fostering knowledge hiding (Xu et al., 2022). Authoritarian leadership increases job demands while eroding resources, thereby intensifying employees’ perceptions of hindrance stressors. These stressors activate a resource conservation process, prompting employees to adopt various knowledge-hiding strategies, including evasive, playing dumb, rationalized, and procrastination-style hiding, to protect themselves from further loss. By limiting knowledge exchanges, employees attempt to maintain a sense of control, reduce their exposure to punitive consequences, and preserve their psychological resources. Therefore, hindrance stressors serve as crucial psychological mechanisms linking authoritarian leadership to knowledge hiding. Furthermore, identifying hindrance stressors as an important boundary condition extends existing theories by demonstrating that contextual barriers can intensify the detrimental effects of authoritarian leadership. This contributes to a more nuanced understanding of when and why authoritarian leadership is likely to erode knowledge-sharing behaviors. Collectively, these insights advance the theoretical landscape by integrating motivational, resource-based, and contextual perspectives, and refining the explanatory mechanisms that connect authoritarian leadership with knowledge-hiding behavior. The results of the study confirmed that employees who work under authoritarian leaders are more likely to experience hindrance stressors, which subsequently heighten their sense of job insecurity. These escalating psychological pressures activate a self-protective response in the form of knowledge hiding. The validated chain mediation model clarifies how authoritarian leadership triggers a layered process of resource depletion that unfolds through stress appraisal and insecurity perceptions, before culminating in knowledge-withholding behaviors. This contribution deepens the theoretical understanding of how negative leadership leads to counterproductive knowledge behaviors and underscores the importance of interconnected psychological processes when examining how leadership shapes employee cognition and action.

Finally, this study draws upon survey data from employees working in Chinese SMEs operating in fast-changing and resource-constrained organizational environments. Authoritarian leadership remains particularly relevant in the Chinese organizational context, where hierarchical traditions and the emphasis on authority have historically shaped leader–subordinate relationships (Farh and Cheng, 2000; Zhang et al., 2015). Its relevance is also evident in Chinese SMEs, where recent empirical research has specifically documented authoritarian leadership and its influence on employee behaviors (Wang et al., 2022). By focusing on this population, this study extends the applicability of research on authoritarian leadership, hindrance stressors, job insecurity, and knowledge hiding to a context that is highly representative in practice and underexamined in the existing literature. Employees in such firms often face rigid hierarchical structures and limited formalized support systems, making them particularly vulnerable to the psychological mechanisms identified in this study. The findings in this study enhance the external validity of the proposed model and offer context-sensitive insights for scholars and practitioners interested in leadership and knowledge management in emerging market organizational settings. At the same time, SMEs depend heavily on employees’ knowledge sharing to compensate for scarce resources and maintain competitiveness. Understanding how authoritarian leadership, hindrance stressors, and insecurity shape knowledge hiding in this context is theoretically important and urgent. By revealing the psychological and behavioral processes triggered by authoritarian leadership, this study supports the advancement of leadership theory and guides organizations to reduce its negative effects. Such knowledge can guide organizations toward cultivating leadership climates that enhance employee well-being and sustainable development.

### 5.2 Practical implications

This study offers several important practical implications for managers and organizations, particularly within Chinese SMEs. First, the findings show that authoritarian leadership consistently triggers hindrance stressors and job insecurity, ultimately increasing employees’ tendencies to engage in all four forms of knowledge hiding. This suggests that managers need to recognize the detrimental effects of highly controlled leadership behaviors on employees’ psychological states and information-sharing behaviors. Organizations should, therefore, invest in leadership development programs that help supervisors adopt supportive communication practices, reduce punitive control, and strengthen relational trust with subordinates.

Second, the results highlight the critical role of hindrance stressors and job insecurity as psychological mechanisms that translate leadership behaviors into counterproductive knowledge withholding. Managers should actively monitor and reduce sources of hindrance stressors such as unclear role expectations, cumbersome procedures, and organizational politics. Strengthening job stability signals, clarifying performance criteria, and improving fairness in managerial decision-making can help alleviate employees’ perceptions of insecurity and prevent self-protective withdrawal behavior.

Third, because knowledge hiding hampers collaboration and innovation, firms should create resource-enriched work environments that buffer employees from resource depletion. Providing access to training, autonomy, and performance feedback can help rebuild psychological resources and reduce the need for protective withholding behaviors. Additionally, establishing team-based knowledge-sharing norms and reward systems may counteract the negative behavioral tendencies triggered by authoritarian leadership.

Overall, these practical implications suggest that reducing hindrance stressors, conveying job stability, and cultivating supportive leadership styles can help Chinese SMEs foster open, cooperative, and innovative organizational climates.

### 5.3 Limitations and future research

This study has several limitations that provide valuable opportunities for future research. First, although the study focused on employees working in Chinese SMEs, detailed organizational information, including industry sector, geographical region, firm size, and sampling frame, was not systematically incorporated into the research design. As a result, the findings should be interpreted within the context of the sampled organizations rather than generalized to the entire population of SMEs in China. Future research could strengthen the external validity and broader applicability of these findings by drawing samples from a wider range of industries and geographical regions, distinguishing between small and medium sized enterprises, and adopting more representative sampling strategies, such as stratified or probability based sampling. Such efforts would further enhance the external validity and generalizability of the findings while providing a more nuanced understanding of the boundary conditions under which authoritarian leadership influences employee outcomes.

Second, because all study variables were measured using self reported questionnaires collected from the same respondents at a single point in time, common method bias (CMB) may represent a potential concern. To evaluate this issue, this study adopted the full collinearity assessment proposed by Kock (2015). The variance inflation factor values for authoritarian leadership (1.381), hindrance stressors (1.635), and job insecurity (1.522) were all substantially below the recommended threshold of 5.0, suggesting that common method bias was unlikely to pose a serious threat to the validity of the findings. Nevertheless, statistical detection alone cannot completely eliminate the possibility of common method bias because it does not address potential bias introduced during data collection. Future research is therefore encouraged to combine procedural and statistical remedies to further strengthen methodological rigor. For example, researchers may adopt multi source or matched survey designs in which employees evaluate leadership behaviors while supervisors assess employee outcomes, or collect data at different time points to introduce temporal separation. In addition, future studies may supplement full collinearity assessment with other statistical techniques, such as Harman’s single factor test, marker variable approaches, or confirmatory factor analysis marker techniques, to provide more comprehensive evidence that common method bias does not materially influence the observed relationships.

Third, this study focused on hindrance stressors and job insecurity as serial mediators that link authoritarian leadership to the four dimensions of knowledge hiding. Although these mechanisms are theoretically grounded, they do not exhaust possible psychological or contextual pathways. Future research may explore additional mediators, such as fear of negative evaluation, relational distrust, emotional exhaustion, and power distance orientation. Researchers may also investigate boundary conditions, such as team climate, leadership structure, or employee resilience, which may weaken or intensify these effects.

Finally, the present model does not incorporate key boundary conditions characteristic of the Chinese organizational context, including high-power distance, strong collectivistic orientations, and the central role of “guanxi” in workplace relationships. These cultural features can meaningfully shape how employees interpret leader behaviors and respond to knowledge-related situations. Future research could examine how these contextual characteristics function as boundary conditions within the model, offering a comprehensive understanding of cultural influences on the link between leadership and employee knowledge behavior.

## 6 Conclusion

This study examines the underlying mechanisms through which authoritarian leadership affects employee knowledge hiding. This study highlights hindrance stressors and job insecurity as serial multiple mediating variables and empirically tests their roles in linking authoritarian leadership with knowledge hiding. Our research offers new insights into the mechanisms underlying knowledge-hiding practices in Chinese SMEs. Specifically, following the extension proposed by Jin et al. (2023), we investigate whether authoritarian leadership serves as a primary driver of employees’ knowledge hiding and broadens the conceptual scope of knowledge hiding by incorporating procrastination.

Our findings identify a clear mechanism of transmission from authoritarian leadership to knowledge hiding. Authoritarian leadership is positively associated with employees’ knowledge hiding, with hindrance stressors and job insecurity functioning as serial mediators of this relationship.

## Data Availability

The datasets presented in this article are not readily available because the datasets generated and/or analyzed during the current study are available from the corresponding author upon reasonable request. Requests to access the datasets should be directed to XJ, soohua1005@gachon.ac.kr.
